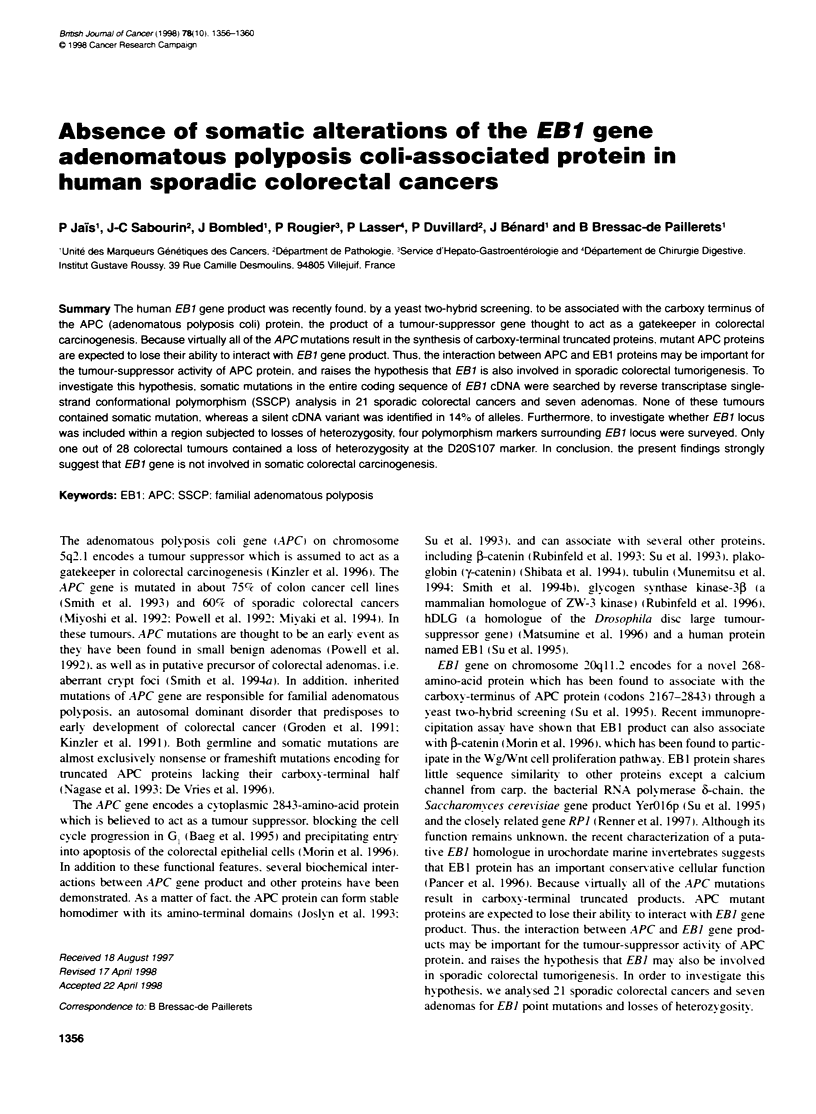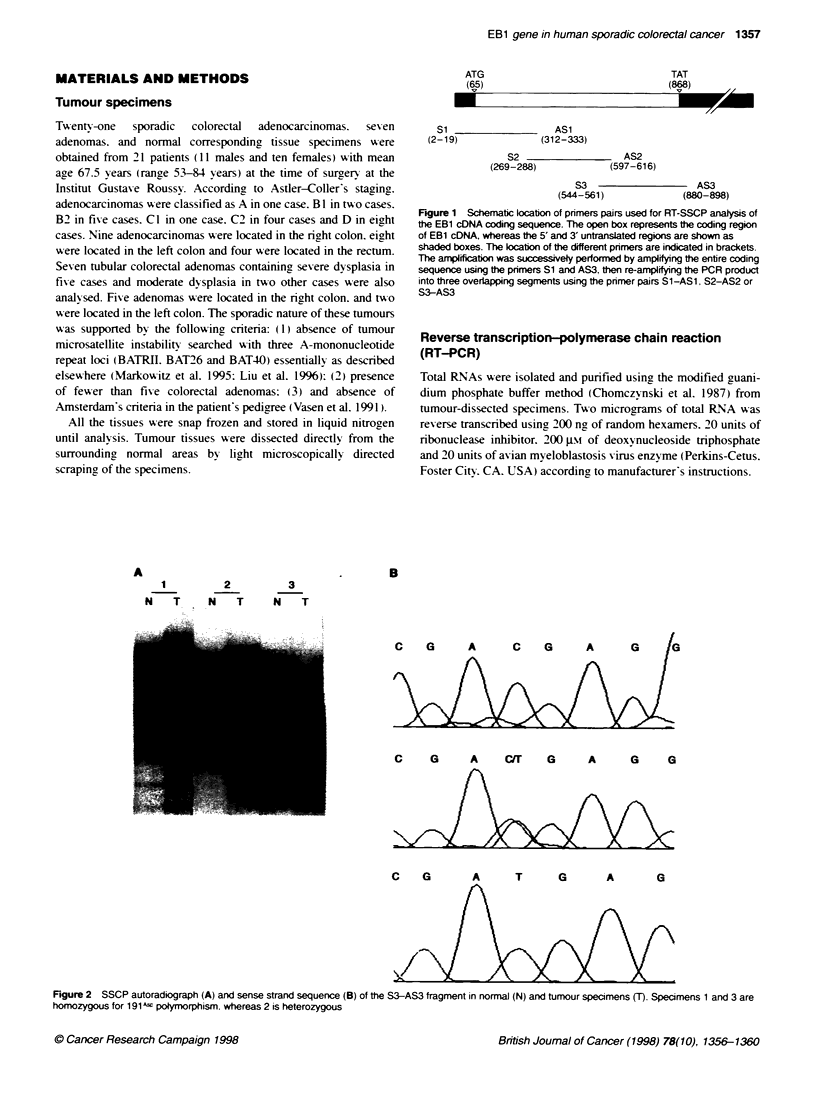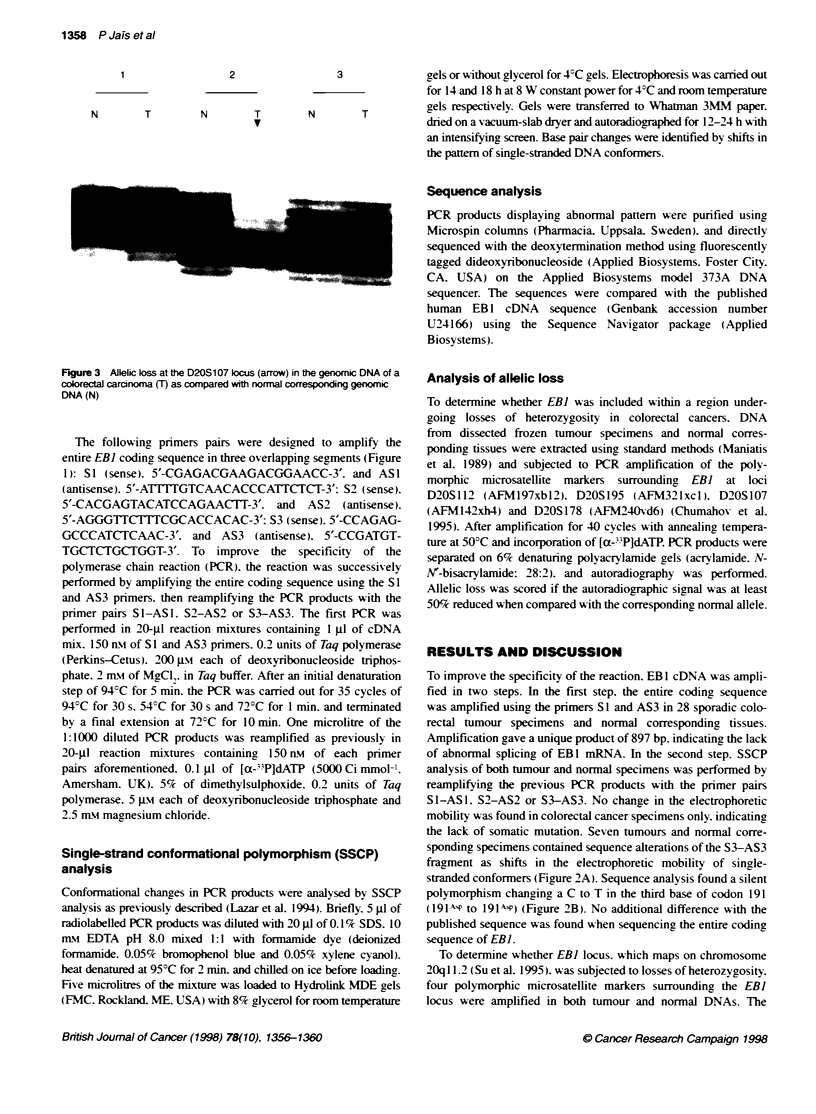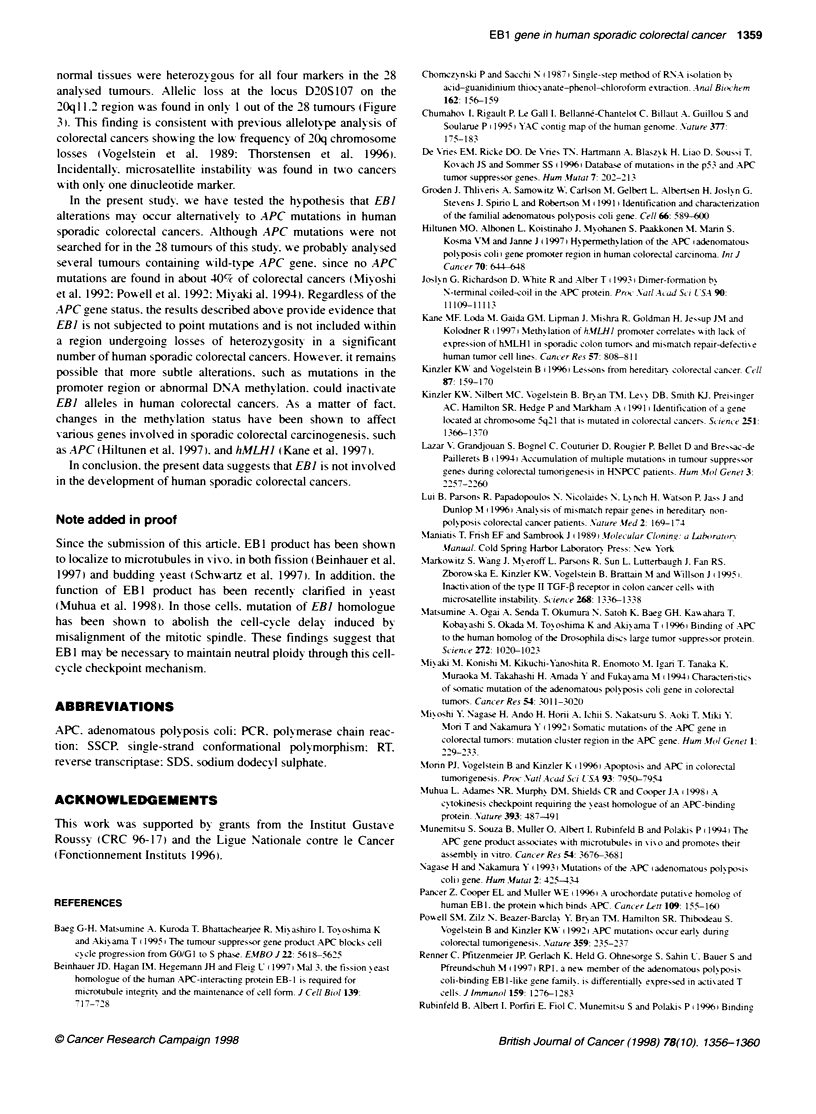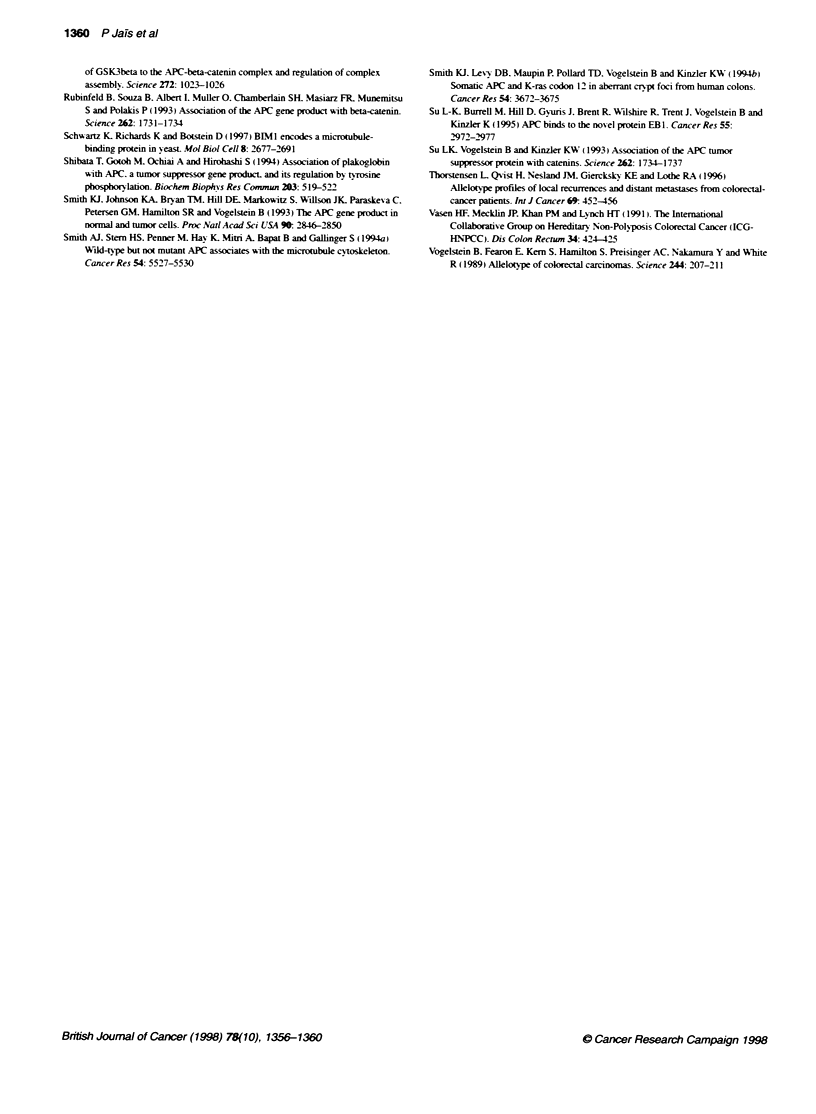# Absence of somatic alterations of the EB1 gene adenomatous polyposis coli-associated protein in human sporadic colorectal cancers.

**DOI:** 10.1038/bjc.1998.684

**Published:** 1998-11

**Authors:** P. Jaïs, J. C. Sabourin, J. Bombled, P. Rougier, P. Lasser, P. Duvillard, J. Bénard, B. Bressac-de Paillerets

**Affiliations:** Unité des Marqueurs Génétiques des Cancers, Institut Gustave Roussy, Villejuif, France.

## Abstract

**Images:**


British Journal of Cancer (1998) 78(10). 1356–1360
© 1998 Cancer Research Campaign

### Absence of somatic alterations of the *EB1* gene adenomatous polyposis coli-associated protein in human sporadic colorectal cancers

**P Jaïs[1], J-C Sabourin[2], J Bombled[1], P Rougier[3], P Lasser[4], P Duvillard[2], J Bénard[1] and B Bressac-de Paillerets[1]**

[1]Unité des Marqueurs Génétiques des Cancers. [2]Départment de Pathologie. [3]Service d'Hepato-Gastroentérologie and [4]Département de Chirurgie Digestive. Institut Gustave Roussy. 39 Rue Camille Desmoulins. 94805 Villejuif. France

**Summary** The human *EB1* gene product was recently found. by a yeast two-hybrid screening. to be associated with the carboxy terminus of the APC (adenomatous polyposis coli) protein. the product of a tumour-suppressor gene thought to act as a gatekeeper in colorectal carcinogenesis. Because virtually all of the *APC* mutations result in the synthesis of carboxy-terminal truncated proteins. mutant APC proteins are expected to lose their ability to interact with *EB1* gene product. Thus. the interaction between APC and EB1 proteins may be important for the tumour-suppressor activity of APC protein. and raises the hypothesis that *EB1* is also involved in sporadic colorectal tumorigenesis. To investigate this hypothesis. somatic mutations in the entire coding sequence of *EB1* cDNA were searched by reverse transcriptase single-strand conformational polymorphism (SSCP) analysis in 21 sporadic colorectal cancers and seven adenomas. None of these tumours contained somatic mutation. whereas a silent cDNA variant was identified in 14% of alleles. Furthermore. to investigate whether *EB1* locus was included within a region subjected to losses of heterozygosity. four polymorphism markers surrounding *EB1* locus were surveyed. Only one out of 28 colorectal tumours contained a loss of heterozygosity at the D20S107 marker. In conclusion. the present findings strongly suggest that *EB1* gene is not involved in somatic colorectal carcinogenesis.

**Keywords:** EB1: APC: SSCP: familial adenomatous polyposis

The adenomatous polyposis coli gene (*APC*) on chromosome 5q2.1 encodes a tumour suppressor which is assumed to act as a gatekeeper in colorectal carcinogenesis (Kinzler et al. 1996). The *APC* gene is mutated in about 75% of colon cancer cell lines (Smith et al. 1993) and 60% of sporadic colorectal cancers (Miyoshi et al. 1992: Powell et al. 1992: Miyaki et al. 1994). In these tumours. *APC* mutations are thought to be an early event as they have been found in small benign adenomas (Powell et al. 1992). as well as in putative precursor of colorectal adenomas. i.e. aberrant crypt foci (Smith et al. 1994*a*). In addition. inherited mutations of *APC* gene are responsible for familial adenomatous polyposis. an autosomal dominant disorder that predisposes to early development of colorectal cancer (Groden et al. 1991: Kinzler et al. 1991). Both germline and somatic mutations are almost exclusively nonsense or frameshift mutations encoding for truncated APC proteins lacking their carboxy-terminal half (Nagase et al. 1993: De Vries et al. 1996).

The *APC* gene encodes a cytoplasmic 2843-amino-acid protein which is believed to act as a tumour suppressor. blocking the cell cycle progression in $G_1$ (Baeg et al. 1995) and precipitating entry into apoptosis of the colorectal epithelial cells (Morin et al. 1996). In addition to these functional features. several biochemical interactions between *APC* gene product and other proteins have been demonstrated. As a matter of fact. the APC protein can form stable homodimer with its amino-terminal domains (Joslyn et al. 1993: Su et al. 1993). and can associate with several other proteins. including β-catenin (Rubinfeld et al. 1993: Su et al. 1993). plakoglobin (γ-catenin) (Shibata et al. 1994). tubulin (Munemitsu et al. 1994: Smith et al. 1994b). glycogen synthase kinase-3β (a mammalian homologue of ZW-3 kinase) (Rubinfeld et al. 1996). hDLG (a homologue of the *Drosophila* disc large tumour-suppressor gene) (Matsumine et al. 1996) and a human protein named EB1 (Su et al. 1995).

*EB1* gene on chromosome 20q11.2 encodes for a novel 268-amino-acid protein which has been found to associate with the carboxy-terminus of APC protein (codons 2167–2843) through a yeast two-hybrid screening (Su et al. 1995). Recent immunoprecipitation assay have shown that EB1 product can also associate with β-catenin (Morin et al. 1996). which has been found to participate in the Wg/Wnt cell proliferation pathway. EB1 protein shares little sequence similarity to other proteins except a calcium channel from carp. the bacterial RNA polymerase δ-chain. the *Saccharomyces cerevisiae* gene product Yer016p (Su et al. 1995) and the closely related gene *RP1* (Renner et al. 1997). Although its function remains unknown. the recent characterization of a putative *EB1* homologue in urochordate marine invertebrates suggests that EB1 protein has an important conservative cellular function (Pancer et al. 1996). Because virtually all of the *APC* mutations result in carboxy-terminal truncated products. APC mutant proteins are expected to lose their ability to interact with *EB1* gene product. Thus. the interaction between *APC* and *EB1* gene products may be important for the tumour-suppressor activity of APC protein. and raises the hypothesis that *EB1* may also be involved in sporadic colorectal tumorigenesis. In order to investigate this hypothesis. we analysed 21 sporadic colorectal cancers and seven adenomas for *EB1* point mutations and losses of heterozygosity.

*Received 18 August 1997*

*Revised 17 April 1998*

*Accepted 22 April 1998*

*Correspondence to:* B Bressac-de Paillerets

### MATERIALS AND METHODS

### Tumour specimens

Twenty-one sporadic colorectal adenocarcinomas, seven adenomas, and normal corresponding tissue specimens were obtained from 21 patients (11 males and ten females) with mean age 67.5 years (range 53–84 years) at the time of surgery at the Institut Gustave Roussy. According to Astler–Coller's staging, adenocarcinomas were classified as A in one case, B1 in two cases, B2 in five cases, C1 in one case, C2 in four cases and D in eight cases. Nine adenocarcinomas were located in the right colon, eight were located in the left colon and four were located in the rectum. Seven tubular colorectal adenomas containing severe dysplasia in five cases and moderate dysplasia in two other cases were also analysed. Five adenomas were located in the right colon, and two were located in the left colon. The sporadic nature of these tumours was supported by the following criteria: (1) absence of tumour microsatellite instability searched with three A-mononucleotide repeat loci (BATRII, BAT26 and BAT40) essentially as described elsewhere (Markowitz et al. 1995; Liu et al. 1996); (2) presence of fewer than five colorectal adenomas; (3) and absence of Amsterdam's criteria in the patient's pedigree (Vasen et al. 1991).

All the tissues were snap frozen and stored in liquid nitrogen until analysis. Tumour tissues were dissected directly from the surrounding normal areas by light microscopically directed scraping of the specimens.

**Figure 1** Schematic location of primers pairs used for RT-SSCP analysis of the EB1 cDNA coding sequence. The open box represents the coding region of EB1 cDNA, whereas the 5′ and 3′ untranslated regions are shown as shaded boxes. The location of the different primers are indicated in brackets. The amplification was successively performed by amplifying the entire coding sequence using the primers S1 and AS3, then re-amplifying the PCR product into three overlapping segments using the primer pairs S1–AS1, S2–AS2 or S3–AS3

### Reverse transcription–polymerase chain reaction (RT–PCR)

Total RNAs were isolated and purified using the modified guanidium phosphate buffer method (Chomczynski et al. 1987) from tumour-dissected specimens. Two micrograms of total RNA was reverse transcribed using 200 ng of random hexamers, 20 units of ribonuclease inhibitor, 200 μM of deoxynucleoside triphosphate and 20 units of avian myeloblastosis virus enzyme (Perkins-Cetus, Foster City, CA, USA) according to manufacturer's instructions.

**Figure 2** SSCP autoradiograph (**A**) and sense strand sequence (**B**) of the S3–AS3 fragment in normal (N) and tumour specimens (T). Specimens 1 and 3 are homozygous for $191^{Asp}$ polymorphism, whereas 2 is heterozygous

**Figure 3** Allelic loss at the D20S107 locus (arrow) in the genomic DNA of a colorectal carcinoma (T) as compared with normal corresponding genomic DNA (N)

The following primers pairs were designed to amplify the entire *EB1* coding sequence in three overlapping segments (Figure 1): S1 (sense), 5′-CGAGACGAAGACGGAACC-3′, and AS1 (antisense), 5′-ATTTTGTCAACACCCATTCTCT-3′; S2 (sense), 5′-CACGAGTACATCCAGAACTT-3′, and AS2 (antisense), 5′-AGGGTTCTTTCGCACCACAC-3′; S3 (sense), 5′-CCAGAG-GCCCATCTCAAC-3′, and AS3 (antisense), 5′-CCGATGT-TGCTCTGCTGGT-3′. To improve the specificity of the polymerase chain reaction (PCR), the reaction was successively performed by amplifying the entire coding sequence using the S1 and AS3 primers, then reamplifying the PCR products with the primer pairs S1–AS1, S2–AS2 or S3–AS3. The first PCR was performed in 20-µl reaction mixtures containing 1 µl of cDNA mix, 150 nM of S1 and AS3 primers, 0.2 units of *Taq* polymerase (Perkins–Cetus), 200 µM each of deoxyribonucleoside triphosphate, 2 mM of $MgCl_2$, in *Taq* buffer. After an initial denaturation step of 94°C for 5 min, the PCR was carried out for 35 cycles of 94°C for 30 s, 54°C for 30 s and 72°C for 1 min, and terminated by a final extension at 72°C for 10 min. One microlitre of the 1:1000 diluted PCR products was reamplified as previously in 20-µl reaction mixtures containing 150 nM of each primer pairs aforementioned, 0.1 µl of $[\alpha\text{-}^{33}P]$dATP (5000 Ci mmol$^{-1}$, Amersham, UK), 5% of dimethylsulphoxide, 0.2 units of *Taq* polymerase, 5 µM each of deoxyribonucleoside triphosphate and 2.5 mM magnesium chloride.

### Single-strand conformational polymorphism (SSCP) analysis

Conformational changes in PCR products were analysed by SSCP analysis as previously described (Lazar et al, 1994). Briefly, 5 µl of radiolabelled PCR products was diluted with 20 µl of 0.1% SDS, 10 mM EDTA pH 8.0 mixed 1:1 with formamide dye (deionized formamide, 0.05% bromophenol blue and 0.05% xylene cyanol), heat denatured at 95°C for 2 min, and chilled on ice before loading. Five microlitres of the mixture was loaded to Hydrolink MDE gels (FMC, Rockland, ME, USA) with 8% glycerol for room temperature gels or without glycerol for 4°C gels. Electrophoresis was carried out for 14 and 18 h at 8 W constant power for 4°C and room temperature gels respectively. Gels were transferred to Whatman 3MM paper, dried on a vacuum-slab dryer and autoradiographed for 12–24 h with an intensifying screen. Base pair changes were identified by shifts in the pattern of single-stranded DNA conformers.

### Sequence analysis

PCR products displaying abnormal pattern were purified using Microspin columns (Pharmacia, Uppsala, Sweden), and directly sequenced with the deoxytermination method using fluorescently tagged dideoxyribonucleoside (Applied Biosystems, Foster City, CA, USA) on the Applied Biosystems model 373A DNA sequencer. The sequences were compared with the published human EB1 cDNA sequence (Genbank accession number U24166) using the Sequence Navigator package (Applied Biosystems).

### Analysis of allelic loss

To determine whether *EB1* was included within a region undergoing losses of heterozygosity in colorectal cancers, DNA from dissected frozen tumour specimens and normal corresponding tissues were extracted using standard methods (Maniatis et al, 1989) and subjected to PCR amplification of the polymorphic microsatellite markers surrounding *EB1* at loci D20S112 (AFM197xb12), D20S195 (AFM321xc1), D20S107 (AFM142xh4) and D20S178 (AFM240vd6) (Chumahov et al, 1995). After amplification for 40 cycles with annealing temperature at 50°C and incorporation of $[\alpha\text{-}^{33}P]$dATP, PCR products were separated on 6% denaturing polyacrylamide gels (acrylamide, *N*-*N′*-bisacrylamide: 28:2), and autoradiography was performed. Allelic loss was scored if the autoradiographic signal was at least 50% reduced when compared with the corresponding normal allele.

### RESULTS AND DISCUSSION

To improve the specificity of the reaction, EB1 cDNA was amplified in two steps. In the first step, the entire coding sequence was amplified using the primers S1 and AS3 in 28 sporadic colorectal tumour specimens and normal corresponding tissues. Amplification gave a unique product of 897 bp, indicating the lack of abnormal splicing of EB1 mRNA. In the second step, SSCP analysis of both tumour and normal specimens was performed by reamplifying the previous PCR products with the primer pairs S1–AS1, S2–AS2 or S3–AS3. No change in the electrophoretic mobility was found in colorectal cancer specimens only, indicating the lack of somatic mutation. Seven tumours and normal corresponding specimens contained sequence alterations of the S3–AS3 fragment as shifts in the electrophoretic mobility of single-stranded conformers (Figure 2A). Sequence analysis found a silent polymorphism changing a C to T in the third base of codon 191 ($191^{Asp}$ to $191^{Asp}$) (Figure 2B). No additional difference with the published sequence was found when sequencing the entire coding sequence of *EB1*.

To determine whether *EB1* locus, which maps on chromosome 20q11.2 (Su et al, 1995), was subjected to losses of heterozygosity, four polymorphic microsatellite markers surrounding the *EB1* locus were amplified in both tumour and normal DNAs. The

© Cancer Research Campaign 1998

normal tissues were heterozygous for all four markers in the 28 analysed tumours. Allelic loss at the locus D20S107 on the 20q11.2 region was found in only 1 out of the 28 tumours (Figure 3). This finding is consistent with previous allelotype analysis of colorectal cancers showing the low frequency of 20q chromosome losses (Vogelstein et al. 1989; Thorstensen et al. 1996). Incidentally, microsatellite instability was found in two cancers with only one dinucleotide marker.

In the present study, we have tested the hypothesis that *EB1* alterations may occur alternatively to *APC* mutations in human sporadic colorectal cancers. Although *APC* mutations were not searched for in the 28 tumours of this study, we probably analysed several tumours containing wild-type *APC* gene, since no *APC* mutations are found in about 40% of colorectal cancers (Miyoshi et al. 1992; Powell et al. 1992; Miyaki al. 1994). Regardless of the *APC* gene status, the results described above provide evidence that *EB1* is not subjected to point mutations and is not included within a region undergoing losses of heterozygosity in a significant number of human sporadic colorectal cancers. However, it remains possible that more subtle alterations, such as mutations in the promoter region or abnormal DNA methylation, could inactivate *EB1* alleles in human colorectal cancers. As a matter of fact, changes in the methylation status have been shown to affect various genes involved in sporadic colorectal carcinogenesis, such as *APC* (Hiltunen et al. 1997), and *hMLH1* (Kane et al. 1997).

In conclusion, the present data suggests that *EB1* is not involved in the development of human sporadic colorectal cancers.

### Note added in proof

Since the submission of this article, EB1 product has been shown to localize to microtubules in vivo, in both fission (Beinhauer et al. 1997) and budding yeast (Schwartz et al. 1997). In addition, the function of EB1 product has been recently clarified in yeast (Muhua et al. 1998). In those cells, mutation of *EB1* homologue has been shown to abolish the cell-cycle delay induced by misalignment of the mitotic spindle. These findings suggest that EB1 may be necessary to maintain neutral ploidy through this cell-cycle checkpoint mechanism.

### ABBREVIATIONS

APC, adenomatous polyposis coli; PCR, polymerase chain reaction; SSCP, single-strand conformational polymorphism; RT, reverse transcriptase; SDS, sodium dodecyl sulphate.

### ACKNOWLEDGEMENTS

This work was supported by grants from the Institut Gustave Roussy (CRC 96-17) and the Ligue Nationale contre le Cancer (Fonctionnement Instituts 1996).

### REFERENCES

Baeg G-H, Matsumine A, Kuroda T, Bhattachearjee R, Miyashiro I, Toyoshima K and Akiyama T (1995) The tumour suppressor gene product APC blocks cell cycle progression from G0/G1 to S phase. *EMBO J* **22**: 5618–5625

Beinhauer JD, Hagan IM, Hegemann JH and Fleig U (1997) Mal 3, the fission yeast homologue of the human APC-interacting protein EB-1 is required for microtubule integrity and the maintenance of cell form. *J Cell Biol* **139**: 717–728

Chomczynski P and Sacchi N (1987) Single-step method of RNA isolation by acid–guanidinium thiocyanate–phenol–chloroform extraction. *Anal Biochem* **162**: 156–159

Chumakov I, Rigault P, Le Gall I, Bellanné-Chantelot C, Billaut A, Guillou S and Soularue P (1995) YAC contig map of the human genome. *Nature* **377**: 175–183

De Vries EM, Ricke DO, De Vries TN, Hartmann A, Blaszyk H, Liao D, Soussi T, Kovach JS and Sommer SS (1996) Database of mutations in the p53 and APC tumor suppressor genes. *Hum Mutat* **7**: 202–213

Groden J, Thliveris A, Samowitz W, Carlson M, Gelbert L, Albertsen H, Joslyn G, Stevens J, Spirio L and Robertson M (1991) Identification and characterization of the familial adenomatous polyposis coli gene. *Cell* **66**: 589–600

Hiltunen MO, Alhonen L, Koistinaho J, Myohanen S, Paakkonen M, Marin S, Kosma VM and Janne J (1997) Hypermethylation of the APC (adenomatous polyposis coli) gene promoter region in human colorectal carcinoma. *Int J Cancer* **70**: 644–648

Joslyn G, Richardson D, White R and Alber T (1993) Dimer-formation by N-terminal coiled-coil in the APC protein. *Proc Natl Acad Sci USA* **90**: 11109–11113

Kane MF, Loda M, Gaida GM, Lipman J, Mishra R, Goldman H, Jessup JM and Kolodner R (1997) Methylation of *hMLH1* promoter correlates with lack of expression of hMLH1 in sporadic colon tumors and mismatch repair-defective human tumor cell lines. *Cancer Res* **57**: 808–811

Kinzler KW and Vogelstein B (1996) Lessons from hereditary colorectal cancer. *Cell* **87**: 159–170

Kinzler KW, Nilbert MC, Vogelstein B, Bryan TM, Levy DB, Smith KJ, Preisinger AC, Hamilton SR, Hedge P and Markham A (1991) Identification of a gene located at chromosome 5q21 that is mutated in colorectal cancers. *Science* **251**: 1366–1370

Lazar V, Grandjouan S, Bognel C, Couturier D, Rougier P, Bellet D and Bressac-de Paillerets B (1994) Accumulation of multiple mutations in tumour suppressor genes during colorectal tumorigenesis in HNPCC patients. *Hum Mol Genet* **3**: 2257–2260

Lui B, Parsons R, Papadopoulos N, Nicolaides N, Lynch H, Watson P, Jass J and Dunlop M (1996) Analysis of mismatch repair genes in hereditary non-polyposis colorectal cancer patients. *Nature Med* **2**: 169–174

Maniatis T, Frish EF and Sambrook J (1989) *Molecular Cloning: a Laboratory Manual*. Cold Spring Harbor Laboratory Press: New York

Markowitz S, Wang J, Myeroff L, Parsons R, Sun L, Lutterbaugh J, Fan RS, Zborowska E, Kinzler KW, Vogelstein B, Brattain M and Willson J (1995). Inactivation of the type II TGF-β receptor in colon cancer cells with microsatellite instability. *Science* **268**: 1336–1338

Matsumine A, Ogai A, Senda T, Okumura N, Satoh K, Baeg GH, Kawahara T, Kobayashi S, Okada M, Toyoshima K and Akiyama T (1996) Binding of APC to the human homolog of the Drosophila discs large tumor suppressor protein. *Science* **272**: 1020–1023

Miyaki M, Konishi M, Kikuchi-Yanoshita R, Enomoto M, Igari T, Tanaka K, Muraoka M, Takahashi H, Amada Y and Fukayama M (1994) Characteristics of somatic mutation of the adenomatous polyposis coli gene in colorectal tumors. *Cancer Res* **54**: 3011–3020

Miyoshi Y, Nagase H, Ando H, Horii A, Ichii S, Nakatsuru S, Aoki T, Miki Y, Mori T and Nakamura Y (1992) Somatic mutations of the APC gene in colorectal tumors: mutation cluster region in the APC gene. *Hum Mol Genet* **1**: 229–233.

Morin PJ, Vogelstein B and Kinzler K (1996) Apoptosis and APC in colorectal tumorigenesis. *Proc Natl Acad Sci USA* **93**: 7950–7954

Muhua L, Adames NR, Murphy DM, Shields CR and Cooper JA (1998) A cytokinesis checkpoint requiring the yeast homologue of an APC-binding protein. *Nature* **393**: 487–491

Munemitsu S, Souza B, Muller O, Albert I, Rubinfeld B and Polakis P (1994) The APC gene product associates with microtubules in vivo and promotes their assembly in vitro. *Cancer Res* **54**: 3676–3681

Nagase H and Nakamura Y (1993) Mutations of the APC (adenomatous polyposis coli) gene. *Hum Mutat* **2**: 425–434

Pancer Z, Cooper EL and Muller WE (1996) A urochordate putative homolog of human EB1, the protein which binds APC. *Cancer Lett* **109**: 155–160

Powell SM, Zilz N, Beazer-Barclay Y, Bryan TM, Hamilton SR, Thibodeau S, Vogelstein B and Kinzler KW (1992) APC mutations occur early during colorectal tumorigenesis. *Nature* **359**: 235–237

Renner C, Pfitzenmeier JP, Gerlach K, Held G, Ohnesorge S, Sahin U, Bauer S and Pfreundschuh M (1997) RP1, a new member of the adenomatous polyposis coli-binding EB1-like gene family, is differentially expressed in activated T cells. *J Immunol* **159**: 1276–1283

Rubinfeld B, Albert I, Porfiri E, Fiol C, Munemitsu S and Polakis P (1996) Binding

© Cancer Research Campaign 1998

of GSK3beta to the APC-beta-catenin complex and regulation of complex assembly. *Science* **272**: 1023–1026

Rubinfeld B. Souza B. Albert I. Muller O. Chamberlain SH. Masiarz FR. Munemitsu S and Polakis P (1993) Association of the APC gene product with beta-catenin. *Science* **262**: 1731–1734

Schwartz K. Richards K and Botstein D (1997) BIM1 encodes a microtubule-binding protein in yeast. *Mol Biol Cell* **8**: 2677–2691

Shibata T. Gotoh M. Ochiai A and Hirohashi S (1994) Association of plakoglobin with APC. a tumor suppressor gene product. and its regulation by tyrosine phosphorylation. *Biochem Biophys Res Commun* **203**: 519–522

Smith KJ. Johnson KA. Bryan TM. Hill DE. Markowitz S. Willson JK. Paraskeva C. Petersen GM. Hamilton SR and Vogelstein B (1993) The APC gene product in normal and tumor cells. *Proc Natl Acad Sci USA* **90**: 2846–2850

Smith AJ. Stern HS. Penner M. Hay K. Mitri A. Bapat B and Gallinger S (1994*a*) Wild-type but not mutant APC associates with the microtubule cytoskeleton. *Cancer Res* **54**: 5527–5530

Smith KJ. Levy DB. Maupin P. Pollard TD. Vogelstein B and Kinzler KW (1994*b*) Somatic APC and K-ras codon 12 in aberrant crypt foci from human colons. *Cancer Res* **54**: 3672–3675

Su L-K. Burrell M. Hill D. Gyuris J. Brent R. Wilshire R. Trent J. Vogelstein B and Kinzler K (1995) APC binds to the novel protein EB1. *Cancer Res* **55**: 2972–2977

Su LK. Vogelstein B and Kinzler KW (1993) Association of the APC tumor suppressor protein with catenins. *Science* **262**: 1734–1737

Thorstensen L. Qvist H. Nesland JM. Giercksky KE and Lothe RA (1996) Allelotype profiles of local recurrences and distant metastases from colorectal-cancer patients. *Int J Cancer* **69**: 452–456

Vasen HF. Mecklin JP. Khan PM and Lynch HT (1991). The International Collaborative Group on Hereditary Non-Polyposis Colorectal Cancer (ICG-HNPCC). *Dis Colon Rectum* **34**: 424–425

Vogelstein B. Fearon E. Kern S. Hamilton S. Preisinger AC. Nakamura Y and White R (1989) Allelotype of colorectal carcinomas. *Science* **244**: 207–211

© Cancer Research Campaign 1998